# The Role of Intellectual Humility, Trauma, and Psychopathy in Gun Violence: Understanding Their Implications on a Path Toward Prevention

**DOI:** 10.7759/cureus.93220

**Published:** 2025-09-25

**Authors:** Maya Shah, Amandeep Dhaliwal

**Affiliations:** 1 Biology, Virginia Commonwealth University, Richmond, USA; 2 Psychology, The University of Texas at Arlington, Arlington, USA

**Keywords:** attachment, childhood trauma, gun violence, intellectual humility, psychopathy

## Abstract

Gun violence has become a threatening phenomenon that has been affecting the lives of Americans nationwide. Individuals exhibiting psychopathic traits have a higher propensity for exhibiting gun violence. Trauma is also a significant factor associated with an increased risk for gun violence. We focused on determining the relationships between intellectual humility (IH), psychopathy, and childhood trauma to understand those predisposed toward gun violence, in hopes of contributing to future preventative measures against gun violence.

A total of 162 participants above the age of 18 completed the Comprehensive Intellectual Humility Scale, Marlowe-Crowne Social Desirability Scale, Levenson Self-Report Psychopathy Scale, and Adverse Childhood Experiences (ACE) Questionnaire. All scales were measured on a Likert scale ranging from 1 (strongly disagree) to 5 (strongly agree). A normality test, correlation analysis, linear regression models, and moderation analysis were performed. When looking at the relationship between these two traits, we found that psychopathy had a positive relationship with trauma (r = 0.75, p < 0.001). This means that there is a highly reactive at-risk population that is traumatized and psychopathic, which is more likely to use guns for violent purposes. A moderation analysis showed that the relationship between trauma and psychopathy was moderated by IH (F(3, 157) = 74.16, p < 0.001). So, increasing IH by systematically teaching the concepts associated with IH in schools could decrease the levels of psychopathy in traumatized individuals. This would lower a key trait that relates to an increased risk of committing an act of violence. We seek to enhance understanding of the relationships between predisposing traits for gun violence and explore IH's impact on these relationships.

## Introduction

During the last 20 years, there has been an increase in mass shootings [1]. The number of mass shootings has increased from approximately 15,000 in 2016 to around 20,000 in 2022 [2]. Not only does gun violence take the lives of innocent bystanders, but it has also been related to an estimated prevalence of 91% for posttraumatic stress disorder and 71% for major depression in communities affected by and survivors of mass shootings [3]. Two main traits related to a predisposition toward gun violence in adolescents and adults are psychopathy [4,5] and trauma [6]. There is a dearth of information on the relationships between psychopathy and trauma in these at-risk individuals. For this study, we focused on how intellectual humility (IH) affects the interaction between childhood trauma and levels of psychopathic traits. We hope to understand the traits that affect one's propensity toward gun violence.

Psychopathy

Psychopathy is a personality disorder characterized by a range of traits such as callousness, manipulativeness, impulsivity, and lack of empathy [7]. Psychopathy has been defined as an indicator of gun violence. A study found that psychopathic traits predict patterns of gun-carrying among a sample of adolescents [8]. Another study found that psychopathic traits were associated with higher levels of reactive aggression, which in turn was associated with a greater likelihood of using a gun to commit a violent act [9]. Additionally, one study found that the presence of psychopathy in incarcerated women increased their likelihood of being related to violent crime, specifically crimes involving guns [5]. Firearm violence was also positively related to many different facets of gun violence [10]. Not only is psychopathy related to gun violence, but it is also an established predisposing factor for violence [11]. Psychopathy can also lead to an increase in reactive aggression due to a lack of standard social techniques [12].

Trauma

Childhood trauma can include multiple types of abuse, neglect, and violence between parents or caregivers; other kinds of serious household dysfunction, such as alcohol and substance abuse; and peer, community, and collective violence [13]. Trauma has been identified as a cause for a predisposition toward gun violence in individuals. Specifically, childhood exposures to domestic and community violence can increase one's future likelihood to commit an act of gun violence [6]. Additionally, challenges such as food insecurity have been shown to relate to an increased predisposition toward gun violence [14]. Childhood trauma has also been related to higher levels of aggression in male prisoners [15].

Intellectual humility

IH is a trait that measures one's ability to accept that they are wrong and change their mind given the evidence [16]. IH is characterized by an openness to new ideas, a willingness to revise one's beliefs, and an ability to recognize one's limitations and fallibility [17]. IH has been negatively correlated to the dark triad as a whole (i.e., Machiavellianism, narcissism, and psychopathy). Machiavellianism is characterized by manipulativeness, cynicism, and emotional coldness. Narcissism is characterized by grandiosity, entitlement, and dominance. Psychopathy is characterized by thrill-seeking, low empathy, and anxiety. However, researchers measured a collective dark triad without explicitly addressing the relationship between IH and psychopathy [18]. Furthermore, the effects of being intellectually humble have not been directly linked to trauma. However, due to IH's relation to increased empathy and prosocial values [16], IH may serve as a possible moderator of psychopathy levels due to its potential reduction of the "callous" and "ruthless" nature of psychopaths.

Trait relations

The relationship between environmental factors (e.g., childhood trauma) and individual differences (e.g., traits such as psychopathy and intellectual humility) can provide insight into understanding the population with a tendency toward gun violence. A study done by Craparo et al. showed that higher levels of childhood trauma were related to higher levels of psychopathy in a group of violent offenders from Italy [19]. However, while psychopathy and trauma are indicators of potential gun violence, not all affected individuals become violent. Attachment Theory illustrates how maladaptive attachment styles can lead to increased aggression, explaining the link between trauma and gun violence.

John Bowlby and Mary Ainsworth's Attachment Theory posits that emotional bonds and relationships develop between individuals, particularly between infants and their caregivers [20]. Childhood trauma can disrupt the formation of secure attachments, leading to difficulty developing empathy and positive relationships. If this trauma occurs at a young age for a child with high levels of psychopathy, the unstable early relationship potentially contributes to the pre-existing violent tendencies [21]. A critical part of Attachment Theory is mentalizing. Mentalization is a process in which a child develops an accurate understanding of both the child's internal perception of psychic and external reality [21]. The child relies on a stable figure in their life to help differentiate "pretend modes" from reality [1]. However, maladaptive attachment to an abusive parent can disrupt the mentalization process, which can lead to cognitive dissonance between one's internal state and external reality, or may cause a misunderstanding of others' intent [21]. Additionally, a study by Taubner et al. found that mentalization serves as a moderator between psychopathy and proactive aggression [21]. If mentalization does not occur in those who have formed insecure attachments (i.e., been traumatized), then the child has nothing to impede their propensity for aggression [21]. These two effects can contribute to a regressive state and a further increase in violence [1].

Since trauma serves as an impeding factor for emotional avenues of lowering psychopathic relation to violence, we want to explore the logical side of those with high levels of psychopathy who have been traumatized. One key concept that relates to one's thought processes is intellectual humility. Research suggests that promoting IH may be a useful strategy for reducing polarization and promoting constructive dialogue on issues related to gun violence [22].

The purpose of this study was to determine the relationships between IH, psychopathy, and childhood trauma to understand those predisposed toward gun violence, in hopes of contributing to future preventative measures against gun violence. We hypothesize that individuals who are traumatized will have a positive relationship with psychopathy. Additionally, we hypothesize that varying levels of intellectual humility would decrease the correlation between trauma and psychopathy. This would mean that higher IH levels would result in decreased psychopathic tendencies in traumatized individuals. We hypothesize that psychopathy and trauma will be negatively correlated with IH. This is because trauma has been theorized to result in regressed individuals, resulting in more violent and volatile individuals [21].

## Materials and methods

Participants

Participants were randomly selected from Amazon MTurk users to complete an online survey distributed via MTurk and created using QuestionPro. In order to have an adequately powered study, 150 participants were needed. We took a sample of 200 participants. Of the 200, 162 individuals fully completed the survey. A compensation of $1.00 was provided to the participants. The participants were required to be 18 years of age and United States citizens. If the participants failed to complete the survey or pass the attention checks, they were no longer considered suitable for the study and were not compensated. This study was conducted from June 21, 2023, to July 21, 2023.

Materials and procedure

Participants consented and completed demographic information questionnaires (age, gender, education level, ethnicity, and political views). This study was approved by The University of Texas at Arlington Institutional Review Board (protocol number: 2023-0321). Participants then completed the 23-item Comprehensive Intellectual Humility Scale [17], with a Cronbach's alpha value of 0.88. The four subscales of the Comprehensive IH Scale are "separating one's ego from one's intellect," "changing one's viewpoint based on others' viewpoints," "respecting others' viewpoints," and "lack of overconfidence" [17]. Participants also completed the 13-item Marlowe-Crowne Social Desirability Scale [23], with a Cronbach's alpha of 0.76. The 26-item Levenson Self-Report Psychopathy Scale [7], with a Cronbach's alpha of 0.82, was used to measure psychopathy. Lastly, the participants completed the Adverse Childhood Experiences (ACE) Trauma Scale [24], with a Cronbach's alpha of 0.88. The 10-item Adverse Childhood Experiences (ACE) Questionnaire was developed to measure the most intense and frequently occurring traumatic experiences that children suffer when they are young. The three subscales of the ACE Questionnaire are abuse, neglect, and household conflict. All scales were measured on a Likert scale ranging from 1 (strongly disagree) to 5 (strongly agree). The Comprehensive Intellectual Humility Scale, Marlowe-Crowne Social Desirability Scale, Levenson Self-Report Psychopathy Scale, and Adverse Childhood Experiences Trauma Scale were all free to use and did not require a license or permission. We did not use any definitive "score interpretation range" for our scales, as there are few definitive ranges in the literature. However, we did use sample percentiles to differentiate "high" versus "low" levels of traits.

A normality test, correlation analysis, linear regression models, and moderation analysis were performed. The linear regression and correlation analyses were run through the JASP software (JASP Team (2025)). The moderation analysis and normality test were done using SPSS software (IBM Corp., Armonk, NY). The correlation analysis was done to provide a general understanding of the trait relationships. The linear regression was done to elucidate the independent effects of IH and trauma on psychopathy before testing for moderating effects. The moderation analysis was done to determine the effect that IH had on the relationship between psychopathy and trauma. A p-value less than 0.05 was considered significant.

## Results

Participant characteristics

There were a total of 162 participants, of whom 64% were men. Of the participants, 10% were Asian, 1% were Black/African, 76% were Caucasian, 2% were Hispanic/Latin, 9% were Native American, and 2% had other ethnicities. The participants had a variety of political views (liberal, 30%; conservative, 58%; and moderate, 12%). The participants' ages ranged between 20 and 60 years (mean = 34.14, standard deviation (SD) = 9.24). The participants had a range of education levels, from no formal education to a doctorate. Most of the population had a bachelor's degree (70.4%).

Correlation analysis

Before the analysis, the test of normality was performed on each continuous variable. The psychopathy scale was normally distributed (skewness = -0.37, kurtosis = 0.43) with no outliers. The Adverse Childhood Experiences (ACE) scale was normally distributed (skewness = -0.82, kurtosis = -0.33), with no outliers. The Comprehensive Intellectual Humility Scale was positively skewed and leptokurtic (skewness = 2.29, kurtosis = 6.10). Further analysis of the Q-Q plot and box-whisker plot indicated several outliers. A log transformation was performed on the IH scale, which corrected for the skewness (1.86). The results were similar with and without outliers; therefore, the reported analysis includes outliers. As per recommendations, the skewness value is within +/- 2, and further statistical analyses were performed using the log-transformed variable [25].

The age of the participants was negatively correlated with trauma (r = -0.38, p < 0.001) and psychopathy (r = -0.35, p < 0.001), while positively correlated with intellectual humility (r = 0.38, p < 0.001). To account for participants' bias in responding to survey questionnaires, we also measured their tendency to respond in a socially desirable manner with the Marlowe-Crowne Social Desirability Scale [26]. Additionally, correlations were run between all IH subscales, psychopathy, and childhood trauma (Table 1).

**Table 1:** Correlations between all trait subsets IH 1: Intellectual Humility Subscale 1, IH 2: Intellectual Humility Subscale 2, IH 3: Intellectual Humility Subscale 3, IH 4: Intellectual Humility Subscale 4, PT: Psychopathy Total, ACE Total: Adverse Childhood Experiences Total, SDT: Social Desirability Total

Variable		IH Total	IH 1	IH 2	IH 3	IH 4	PT	ACE Total	SDT
IH 1	Pearson's r	0.689	-						
	p-value	<0.001	-						
IH 2	Pearson's r	0.382	-0.221	-					
	p-value	<0.001	0.005	-					
IH 3	Pearson's r	0.474	-0.124	0.599	-				
	p-value	<0.001	0.117	<0.001	-				
IH 4	Pearson's r	0.616	0.628	-0.274	-0.220	-			
	p-value	<0.001	<0.001	0.005	0.006	-			
PT	Pearson's r	-0.498	-0.726	0.373	0.214	-0.724	-		
	p-value	<0.001	<0.001	<0.001	0.006	<0.001	-		
ACE Total	Pearson's r	-0.560	-0.603	0.121	-0.010	-0.594	0.735	-	
	p-value	<0.001	<0.001	0.124	0.898	<0.001	<0.001	-	
SDT	Pearson's r	0.214	0.348	-0.237	-0.002	0.255	-0.331	-0.300	-
	p-value	0.006	<0.001	0.002	0.984	0.001	<0.001	<0.001	-

Psychopathy and trauma

**Figure 1:**
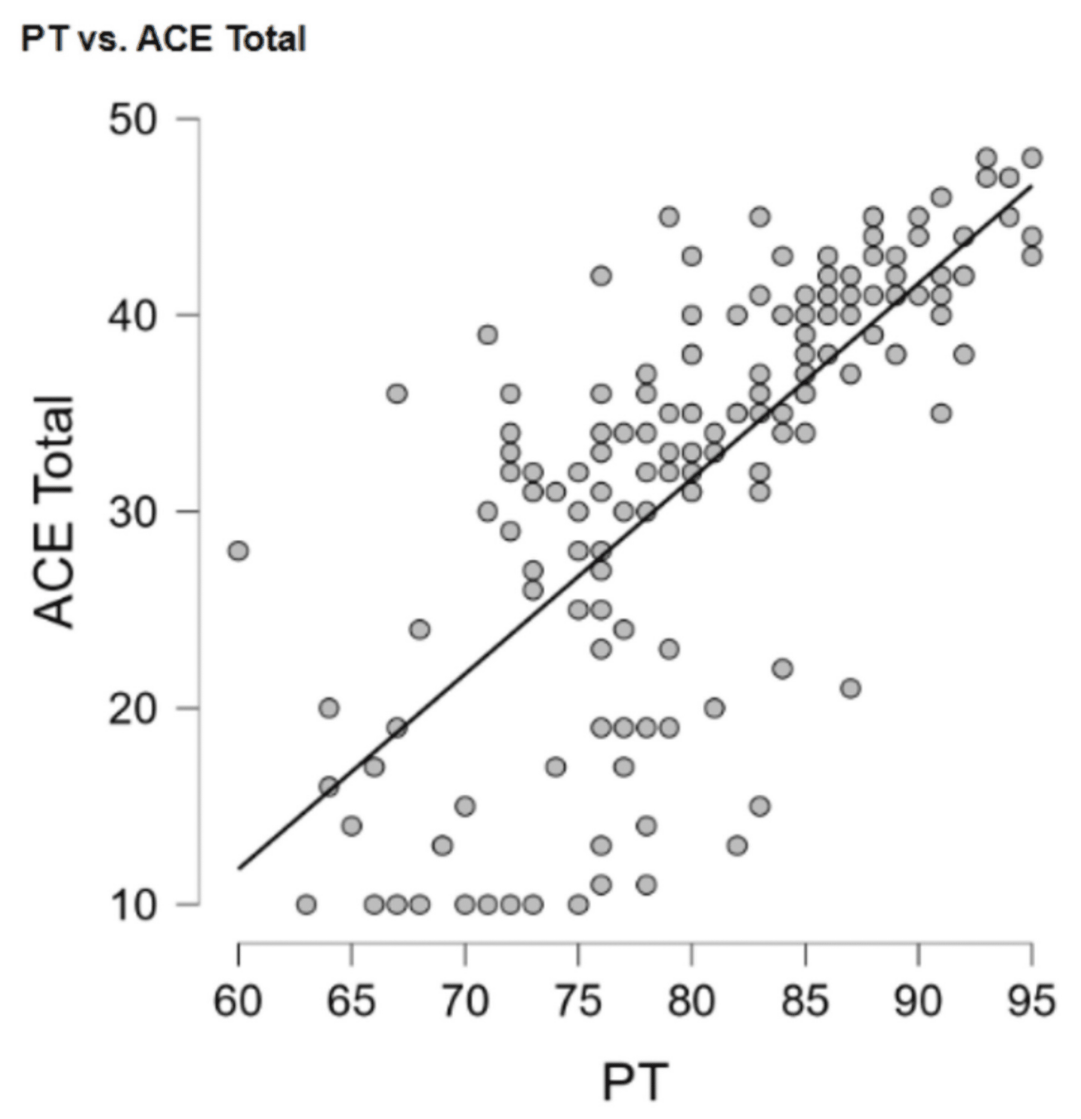
Positive relationship between psychopathy and adverse childhood experiences PT: Psychopathy Total, ACE Total: Adverse Childhood Experiences Total

Psychopathy had a positive relationship with trauma (r = 0.75, p < 0.001) (Figure 1). Additionally, psychopathy had a positive relationship with the three subscales of trauma, abuse (r = 0.03, p = 0.69), neglect (r = 0.12, p = 0.13), and household conflict (r = 0.17, p = 0.04).

Regression analysis

A hierarchical multiple linear regression was performed to account for the role of intellectual humility and trauma on psychopathy while controlling for age and social desirability. We found that after controlling for age and social desirability, childhood trauma and intellectual humility significantly predicted psychopathy (F(4, 156) = 56.87, p < 0.001). Traumatic childhood experiences (𝛽 = 0.61, t(159) = 9.57, p < 0.001) predicted psychopathy, such that for every one unit increase in traumatic experiences, there was a 0.61 unit increase in the psychopathy score. Furthermore, intellectual humility predicted psychopathy scores (𝛽 = -0.15, t(159) = -2.31, p < 0.001), such that for every one unit increase in intellectual humility, there was a decrease of 0.15 units of the psychopathic score.

Moderation analysis

**Figure 2:**
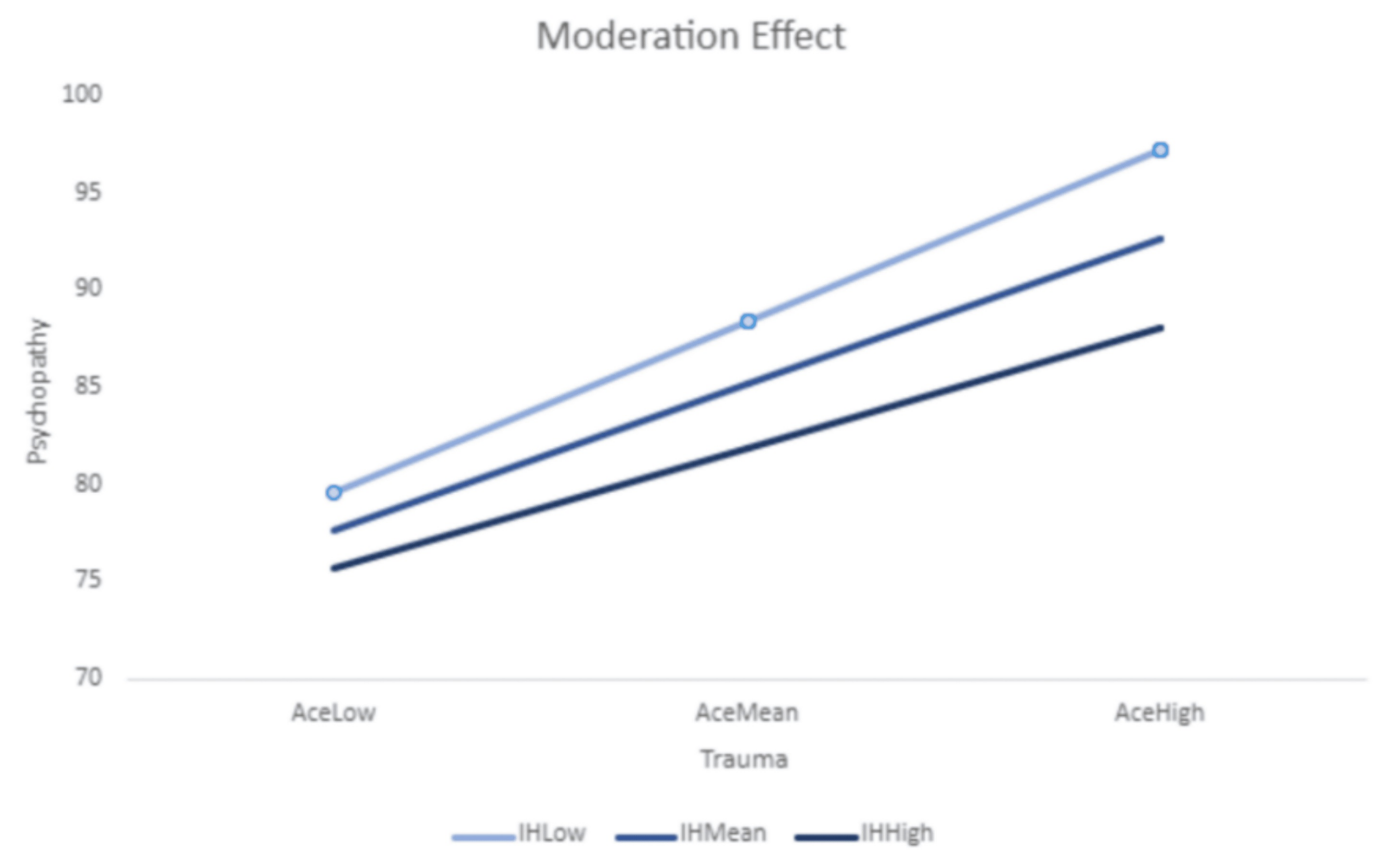
Moderation analysis with intellectual humility as a buffer between childhood trauma and psychopathy

A moderation analysis (using PROCESS v4.2 (https://www.processmacro.org/index.html)) was performed to check if different levels of intellectual humility influence the relationship between trauma and psychopathy. The interaction between trauma and intellectual humility was statistically significant (F(3, 157) = 74.16, p < 0.001). The interaction was probed by testing the conditional effects of intellectual humility at the three levels of trauma, one standard deviation below the mean, at the mean, and one standard deviation above the mean. The results indicate that at low levels of trauma, the psychopathy scores were similar for all three levels of intellectual humility. However, at high levels of trauma, those with higher levels of intellectual humility had significantly lower psychopathy scores than individuals with lower intellectual humility (p < 0.001). We conclude that the moderator (IH) has a significant moderating effect on the relationship between trauma and psychopathy (Table 2, Figure 2).

**Table 2:** Values of psychopathy at different childhood trauma levels based on varying levels of intellectual humility ACE: Adverse Childhood Experiences, IH: intellectual humility

Moderation analysis	Low IH	Mean IH	High IH
Low ACE	79.64	77.72	75.79
Mean ACE	88.49	85.23	81.97
High ACE	97.35	92.75	88.14

IH

**Figure 3:**
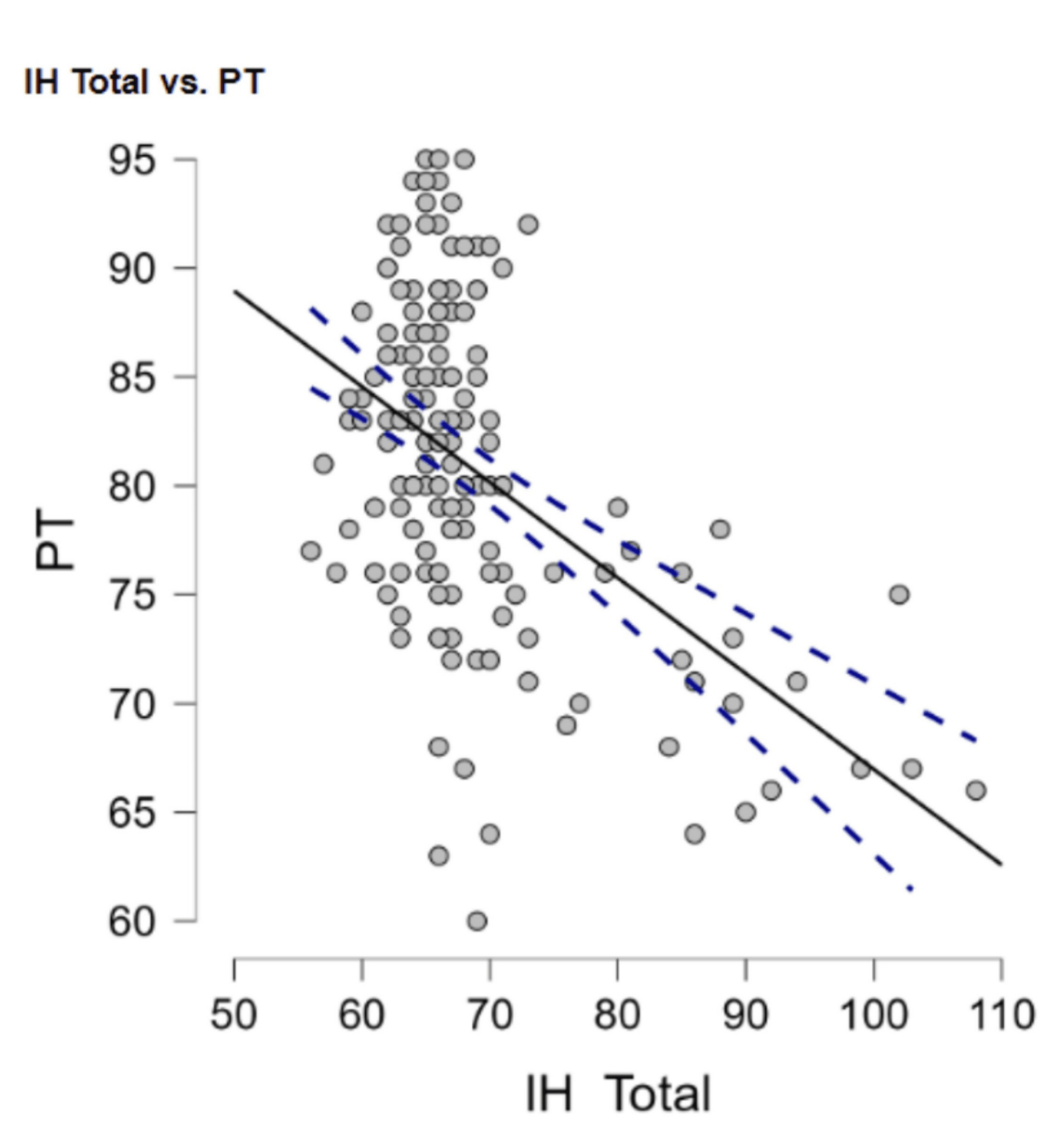
Negative relationship between intellectual humility and psychopathy as a whole PT: Psychopathy Total, IH Total: Intellectual Humility Total

**Figure 4:**
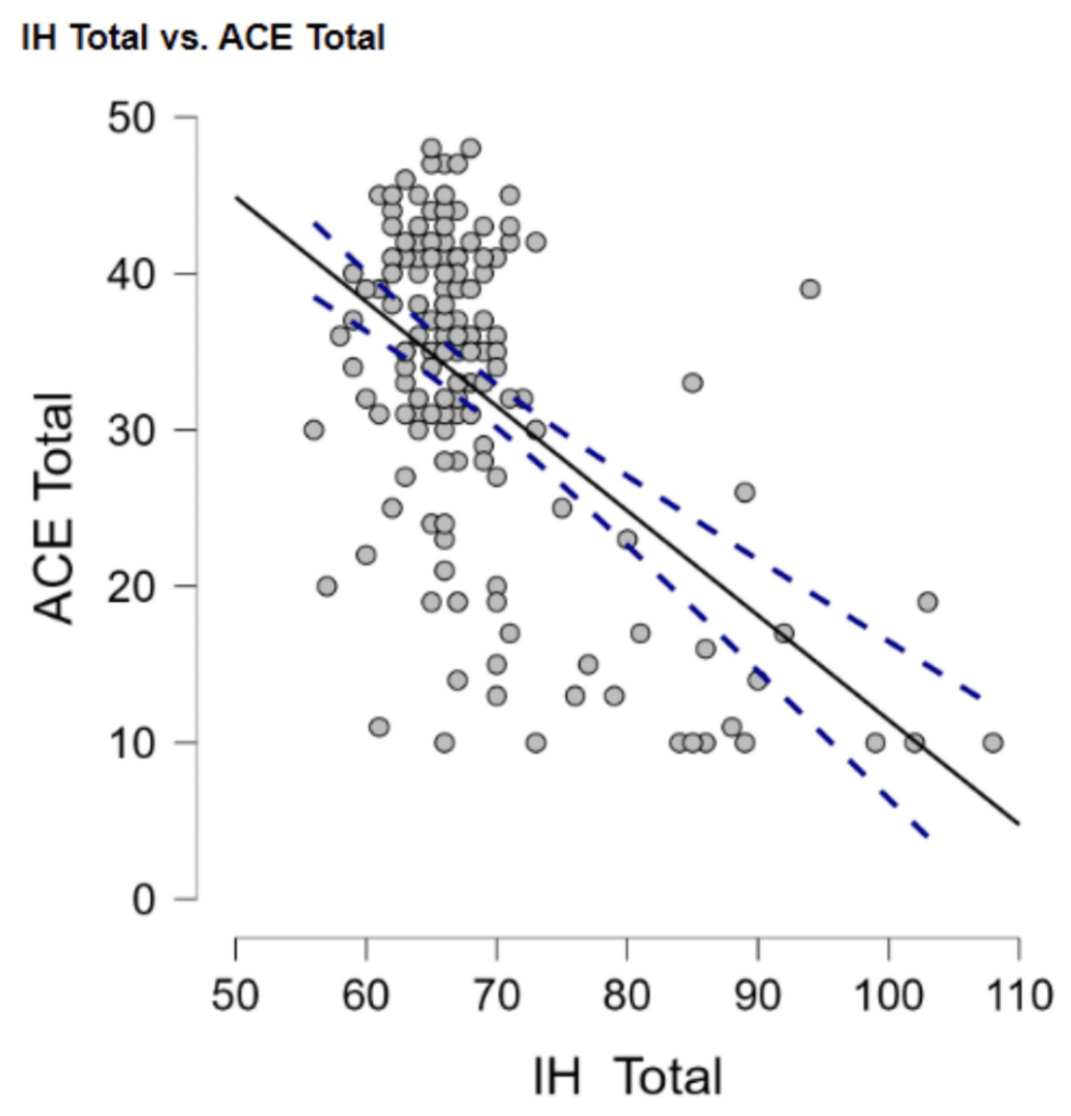
Negative relationship between intellectual humility and adverse childhood experiences ACE Total: Adverse Childhood Experiences Total, IH Total: Intellectual Humility Total

Intellectual humility as a whole had a moderately negative relationship with psychopathy (r = -0.51, p < 0.001) (Figure 3). Intellectual humility also had a moderately negative relationship with trauma (r = -0.54, p < 0.001) (Figure 4).

## Discussion

Pre-existing literature points to a propensity for gun violence in psychopathic individuals [8,9]. Not only is trauma an indicator of gun violence, but it is also a key predisposing factor for general violence. Trauma is related to future violence [27]. This could be due to the decrease in trust and increased volatility that trauma causes [28]. Interventions against child violence should consider trauma in their approach [29]. Psychopathy is a character trait that changes throughout life [30]. In contrast, trauma is something that cannot be changed in individuals. So, traumatized individuals with psychopathy are violent rather than the other way around. This study is unique because it not only supports the idea that psychopathy and trauma are risk factors for gun violence, but it also provides novel information on how IH relates to these two risk factors. It is also the first study to analyze the relationship between IH, psychopathy, and trauma. Additionally, it looks at these trait relations in the context of gun violence.

Some limitations of this study include the cohort size and the age of the participants. This study had a population size of 162. Having a larger population when replicating this study could provide more accurate estimates and create better generalizability. Additionally, while the relationships between IH, psychopathy, and trauma have been explored in this study among adults 18 and older, studying these traits in children could contribute further to the literature. While IH's moderating effect could have a mitigating effect on psychopathy, other environmental factors could also affect the phenotypical development of psychopathy. Especially in children, environmental exposures, such as stress regulation and social interactions, can affect the development of psychopathy. This may moderate the effect of our observed outcomes.

The findings confirm that psychopathy has a positive relationship with trauma. This means that psychopathic individuals are often traumatized. These individuals may be more likely to commit an act of gun violence, as illustrated through attachment theory. The psychopathy component in traumatized individuals could be a reason why certain traumatized persons commit the act of gun violence. So, this indicates the existence of a population of at-risk individuals who are highly prone to gun violence that needs to be reached. The findings confirm our hypothesis that psychopathic traits have an inverse relationship with intellectual humility. This further supports the idea that increasing IH could have the opposite effect of decreasing psychopathy. The findings also confirm our hypothesis that trauma would negatively affect intellectual humility. Since those who are traumatized also tend to have psychopathic traits, the methods needed to reach psychopathic individuals will also work on traumatized individuals who are prone to gun violence. 

Our study was adequately powered with a sufficient sample size. Additionally, validated scales were used to accurately measure the characteristics of intellectual humility, psychopathy, social desirability, and childhood trauma. Also, our study's results were strengthened by the control of social desirability for our statistical analyses. Our study provides novel information on the relations between IH, psychopathy, and trauma. It contributes to the literature on gun violence prevention through the novel approach of focusing on the mitigation of gun violence risk factors. This research can contribute to the creation of preventative measures that can lessen gun violence nationwide.

Previous research has effectively increased intellectual humility in middle school students [31]. Specifically, incorporating the ideal of "learning for learning" was shown to help increase the expression of IH in these pre-adolescent children. Future research could investigate the real-world effects of increasing IH in school populations. Students in IH courses could be followed over the course of a year, and IH, psychopathy, and trauma levels could be measured. This study could further explore the real-world impact of the growth of IH on psychopathy and trauma. Additionally, the relationship between trauma and psychopathy in the context of gun violence could be further explored in future studies. Additionally, there's an absence of studies that analyze the relationship between traumatized individuals with and without IH and the effects on psychopathic levels. This could also be further studied. This study could be improved upon with a larger sample size and an in-person population. Ideally, the actual effect of growing IH in children could be measured over the course of a school year to determine IH's true impact on psychopathy levels in a real-world setting.

## Conclusions

The central focus of this study was the moderation analysis, which revealed that intellectual humility reduces the levels of psychopathy in traumatized individuals. This finding indicates that IH serves as an effective buffer, particularly at higher levels of trauma, by decreasing psychopathic tendencies. This supported our initial hypothesis that increased IH would decrease psychopathy levels in traumatized individuals; hence, teaching intellectual humility to traumatized individuals at an early age could decrease the development of psychopathic tendencies in children. This lowers a key predisposing factor for gun violence and diminishes the reactivity of a highly at-risk population of traumatized individuals exhibiting psychopathic tendencies.

These promising results could be used in the future to analyze methods to intervene against aggressive behaviors such as gun violence in schools. Systematic implementation of IH courses in schools could lessen the impact of psychopathy on functioning in traumatized individuals. IH courses include systematic implementation of projects and teaching styles that emphasize passion for information rather than external benefits, like scores and performance metrics. This lessens a key factor for gun violence and could potentially result in a lower likelihood of committing an act of violence; so, insights from studying IH in individuals prone to gun violence could enhance our understanding of psychopathy and trauma as predisposing factors. By applying these findings in the real world, we might develop targeted interventions to mitigate these factors, potentially contributing to a reduction in gun violence over time.
